# Predictors of Dropout Among Psychosomatic Rehabilitation Patients During the COVID-19 Pandemic: Secondary Analysis of a Longitudinal Study of Digital Training

**DOI:** 10.2196/43584

**Published:** 2023-11-27

**Authors:** Lingling Gao, Franziska Maria Keller, Petra Becker, Alina Dahmen, Sonia Lippke

**Affiliations:** 1 Health Psychology and Behavioural Medicine Constructor University Bremen Bremen Germany; 2 Dr. Becker Klinikgruppe Cologne Germany; 3 Klinikum Wolfsburg Wolfsburg Germany

**Keywords:** dropout, web-based study, digital therapy, medical rehabilitation, digital training, mental disorder, psychosomatic rehabilitation, COVID-19

## Abstract

**Background:**

High dropout rates are a common problem reported in web-based studies. Understanding which risk factors interrelate with dropping out from the studies provides the option to prevent dropout by tailoring effective strategies.

**Objective:**

This study aims to contribute an understanding of the predictors of web-based study dropout among psychosomatic rehabilitation patients. We investigated whether sociodemographics, voluntary interventions, physical and mental health, digital use for health and rehabilitation, and COVID-19 pandemic–related variables determine study dropout.

**Methods:**

Patients (N=2155) recruited from 4 psychosomatic rehabilitation clinics in Germany filled in a web-based questionnaire at T1, which was before their rehabilitation stay. Approximately half of the patients (1082/2155, 50.21%) dropped out at T2, which was after the rehabilitation stay, before and during which 3 voluntary digital trainings were provided to them. According to the number of trainings that the patients participated in, they were categorized into a comparison group or 1 of 3 intervention groups. Chi-square tests were performed to examine the differences between dropout patients and retained patients in terms of sociodemographic variables and to compare the dropout rate differences between the comparison and intervention groups. Logistic regression analyses were used to assess what factors were related to study dropout.

**Results:**

The comparison group had the highest dropout rate of 68.4% (173/253) compared with the intervention groups’ dropout rates of 47.98% (749/1561), 50% (96/192), and 42.9% (64/149). Patients with a diagnosis of combined anxiety and depressive disorder had the highest dropout rate of 64% (47/74). Younger patients (those aged <50 y) and patients who were less educated were more likely to drop out of the study. Patients who used health-related apps and the internet less were more likely to drop out of the study. Patients who remained in their jobs and patients who were infected by COVID-19 were more likely to drop out of the study.

**Conclusions:**

This study investigated the predictors of dropout in web-based studies. Different factors such as patient sociodemographics, physical and mental health, digital use, COVID-19 pandemic correlates, and study design can correlate with the dropout rate. For web-based studies with a focus on mental health, it is suggested to consider these possible dropout predictors and take appropriate steps to help patients with a high risk of dropping out overcome difficulties in completing the study.

## Introduction

### Background

Since the start of the COVID-19 pandemic, there has been a sharp increase in web-based research in many areas, including social science [1,2]. Digital research methodologies such as web-based data collection and digital intervention delivery have been conducted broadly [3,4]. The strengths of these digital strategies have been proven to not only overcome difficulties caused by distancing rules but also make up for the shortage of health care professionals as well as provide flexibility for participants and researchers [5,6]. However, the dropout rates were found to be high in many web-based longitudinal studies, with dropout rates of >50% in some cases [1,7]. Patients who withdraw before completing the study are considered to have *dropped out* of the study. These patients cannot be followed at all measurement time points, which leaves missing values in the analysis and in the subsequent comparison of outcomes over time. A high dropout rate may imperil the validity of longitudinal studies and limit the generalizability of the findings [1,8,9]. To explore possible approaches to decreasing the dropout rate, it is essential to identify the factors that relate to whether participants drop out of a web-based study or continue to participate.

According to social cognitive theory, the dropout behavior of participants is related to personal and environmental factors [10]. Personal factors (eg, sociodemographics) were the most consistently targeted predictors of dropout in previous studies and have shown mixed findings [7,11-13]. In addition, physical health and mental health were also possible personal factors that predicted dropout [14,15]. External factors (eg, the digital interventions) could create barriers for participants who have difficulties using digital offerings [7]. According to the health action process approach [16], participants’ cognitive factors, such as intention to use digital offerings, were affected by barriers, which, in turn, related to dropout behavior. Besides, participants faced more challenges during the COVID-19 pandemic period. If participants were infected by COVID-19, they could drop out of the study owing to their worse health status. In the following subsections, these potential predictors of dropout and previous related research are summarized to explain the focus of our work.

### Sociodemographic Predictors of Dropout

Sociodemographics are essential components in social, behavioral, and clinical medical research. If researchers could know which population is at a high risk of dropping out of the study, they could develop solutions that fit the requirements of participants to reduce the dropout rate. Thus, sociodemographics have been investigated by a multitude of studies as the predictors of the dropout rate.

Although female were found to be more likely to engage in the interventions and remain in some research [17-19], they were also found to have a more difficult time remaining in other research owing to fewer expectations or self-efficacy in using a digital service [7,11,12]. Regarding age, older participants were found to be more likely to remain in the study than younger participants [20,21]; this could be because older participants have more trust in service providers in studies [22]. By contrast, other studies argue that older participants who have barriers to using technology were more likely to drop out of the study [23]. Participants’ education level has shown relatively consistent results in previous studies: participants with a higher education level were more likely to remain in the study than those who were less educated [24,25]. Studies also found that participants who were unemployed, those with a low income, and those who were single were more likely to drop out [7,26,27]. However, other studies indicated that there was no significant relationship between dropout rates and 1 or several demographics [1,20,21].

### Health Predictors of Dropout

Health status, including physical health and mental health, are possible predictors of dropout. In a few studies, health behaviors such as regular physical activity and healthy diet were found to make a significant difference when comparing participants who dropped out and those who remained in the study [28]. However, in other studies, this was not seen [14,29]. Participants who reported poor physical health might have been more likely to be limited in their participation during the follow-up measurement time points owing to ill-health [14]. However, some studies found that worse physical health predicted decreased dropout (ie, lower attrition) [13], perhaps because the participants perceived more need to receive attention and support. By contrast, some other studies have shown that it was not physical health status but mental health status that predicted dropout [15]. Participants experiencing mental health problems such as anxiety and depression [30,31] were more likely to drop out of the study because they could have difficulties (eg, motivational deficits) in completing the surveys.

### Other Study Design Correlates: Intervention, Digital Use, and the COVID-19 Pandemic

The mixed findings from different studies might have arisen because of the differences in study designs, which determined not only who could participate but also study processes. Some studies reported that the dropout rate was higher in the intervention group (IG) than in the comparison group (CG), and this was a pattern in web-based studies that conducted internet-based interventions [1,32]. Participants in the IGs in some studies received extra training or services, which imposed an additional assignment burden on the IG participants and therefore increased the chances of dropout [30]. Moreover, mental health clinical studies found that patient preferences (eg, treatment, therapist, and activity preferences) were negatively related to dropout rates [33]. Patients who received the preferred treatment had lower chances of dropping out [34]. It was also found that studies with large samples reported higher dropout rates, and studies offering feedback to participants reported lower dropout rates [1].

High dropout rates were frequently reported in web-based studies, despite the convenience that digital use in the studies provided to both the research and participants [35,36]. Most internet-based intervention studies, require participants to have prior experience using digital devices such as computers and mobile phones with access to the internet [37]. Digital health literacy, attitude, intention, and the ability to make positive use of digital offerings were found to be related to survey completion rates [23,38,39].

Although web-based studies play an important role, especially since the breakout of the COVID-19 pandemic, there are both positive and negative aspects to how the pandemic-related factors predict dropout. On the one hand, people have more expectations of, and higher intention to participate in, web-based studies and digital interventions owing to the inconvenience caused by COVID-19–related restrictions (eg, physical distancing rules). On the other hand, the negative effects associated with the COVID-19 pandemic (eg, a worse health status owing to COVID-19 infection) may be related to study dropout rates. How factors such as worries about losing one’s job, the consequent financial impacts, and anxiety owing to the circumstances of the pandemic relate to dropout are still unknown.

### Research Questions

This study aims to examine what factors are associated with digital study dropout among patients with mental health issues. Specifically, it aims to answer the following research questions:

1. What are the differences in characteristics between patients who dropped out and those who remained in the study?
2. Do differences exist between the IGs and CG regarding dropout?
3. What factors are related to completing the study from the perspectives of health, digital use, and the COVID-19 pandemic correlates?
4. Do differences exist between retained patients and dropout patients regarding the time spent answering the questionnaire at baseline?

## Methods

### Ethical Considerations

Information regarding this study was provided on the portal of the rehabilitation clinics (which supported our study) and could be viewed by patients who had access to the portal with an individualized participant code. If participants expressed a desire to participate in the study, they were informed about the study conditions. Participants who signed the written informed consent before the start of rehabilitation were included in the study. The study data were pseudonymized. No compensation was offered to patients for their participation in this study.

The web-based survey received ethics approval from the ethics committee at Jacobs University in Bremen, Germany (2020_09; June 25, 2020). This study was conducted as part of the project “Anhand-COVID19-Offer to achieve treatment and rehabilitation goals in compliance with hygiene and social-distancing rules” (ClinicalTrials.gov: NCT04453475), which is supported by Dr Becker Klinikgruppe. The data are stored on secure servers at Constructor University (formerly Jacobs University, which began operating as Constructor University in November 2022).

### Interventions and Recruitment

Three digital trainings were provided in this study (Textbox 1).

**Digital trainings offered on a voluntary basis in this study.**

**Rehabilitation goals digital training**

- This was offered to all patients before the beginning of the rehabilitation stay. This training instructed patients to understand why it is important to formulate goals and plans and how to formulate their own rehabilitation goals and plans. It was conducted once using a digital PowerPoint (Microsoft Corp) presentation used for educative content and a digital exercise booklet used for interactive content. Patients could participate in this training at home via the internet.

**Digital group training for depression**

- This was only for patients who had been diagnosed with major depression. In this training, patients learned the symptoms of depression, coping mechanisms, and available treatments for depression. The training included 6 digital group therapy sessions, with each session consisting of a 5-min digital training followed by a 45-min analog group session. A therapist with a flip chart accompanied patients in each session.

**Informative digital training on the legal rights of people who are (severely) disabled**

- This was provided to all patients in a group session format during their rehabilitation stay. In this training, patients got to know the law on severe disability, the requirements for obtaining a certificate of disability, and its consequences on everyday life. It was conducted once with a 20-min informative video followed by a 25-min face-to-face group session to discuss in-depth questions.

This longitudinal study recruited participants from 4 psychosomatic rehabilitation clinics operated by Dr Becker Klinikgruppe in Germany. Data were collected between July 1, 2020, and June 30, 2021. The first measurement time point (T1) was at 6 weeks before the rehabilitation stay until the first day of the rehabilitation stay. The second measurement time point (T2) was after the rehabilitation stay (a maximum of 12 wk after rehabilitation, with 3 reminders to complete the questionnaire sent 1, 4, and 11 wk after the rehabilitation stay).

A total of 2155 participants were recruited at baseline. Of the 2155 participants, 1397 (64.83%) were female, 1209 (56.1%) were aged ≥50 years, and 1571 (72.9%) had a vocational training or university degree.

### Measures

Sociodemographic information on sex, age, and education level was collected via a web-based questionnaire and measured as categorical variables.

Rehabilitation goals were measured by an 8-item scale (consisting of the possible rehabilitation goals that patients aimed to achieve during the rehabilitation) that has shown acceptable reliability and validity [40]. Sample items included “Improvement of my flexibility and endurance.” Answers were rated on a 4-point Likert scale ranging from 1 (not at all) to 4 (completely). Cronbach α values were .69 at T1 and .90 at T2 in this study.

Physical health status and mental health status were assessed from the following aspects. Regular physical activity behavior was measured by asking, “How often were you physically active for 30 minutes or more, for example doing sports?” Diet behavior was measured by asking, “Did you eat five servings of vegetables and fruit daily?” These 2 measures of health behaviors have shown acceptable reliability and validity in previous studies [41]. BMI (kg/m^2^) was calculated using self-reported height and weight and categorized as underweight, normal weight, overweight, and obese [42]. Preexisting illness was measured by asking, “Do you have underlying diseases such as cardiovascular diseases, diabetes, diseases of the respiratory system, liver and kidney diseases and cancer?” Possible disability status was measured by asking, “Do you hold a severely disabled person’s pass?” The disability and health risk factor (preexisting illness) measures have shown acceptable reliability and validity in previous studies [43]. Perceived loneliness was measured by 2 items: “How often do you feel lonely?” [44] “How often do you feel unhappy to be alone?” [45]. The answers were rated on a 4-point Likert scale ranging from 1 (not at all) to 4 (almost every day), with Spearman ρ values of 0.82 at T1 and 0.84 at T2 in this study. Perceived stress was measured by the short 4-item version of the Perceived Stress Scale (PSS-4) [46], with answers rated on a 5-point Likert scale ranging from 0 (never) to 4 (very often) and with Cronbach α values of .74 at T1 and .85 at T2 in this study. Perceived depression and anxiety symptoms were measured by the Patient Health Questionnaire-4 (PHQ-4) [47], with answers rated on a 4-point Likert scale ranging from 0 (not at all) to 3 (nearly every day) and with Cronbach α values of .86 at T1 and .89 at T2 in this study. These aspects were analyzed separately (refer to the *Results* section).

Digital use for health and rehabilitation was assessed from the following aspects. Health-related digital use behavior was measured by asking, “For which health topics do you use apps or the internet?” A total of 11 answers were provided (eg, “exercise and fitness [eg, pedometer]”). This question was adapted and modified based on a systematic review [48] and a survey [49] regarding health app use. Furthermore, two questions measured the stage of health app use: (1) “Do you have an app that helps you communicate well with healthcare professionals, or would you use such an app in the future?” (stage of app 1) and (2) “Do you use a so-called ‘Corona data donation app’ that forwards your data such as resting heart rate, sleep and activity level to scientific institutions and that may show you a warning if you have infected yourself, or would you use such an app?” (stage of app 2). Both questions were answered on a 5-point Likert scale ranging from 1 (no, I don’t intend to) to 5 (yes, and that is very easy for me). Attitude toward digital offerings was measured by asking, “I think the digital offerings would help me a lot,” with answers rated on a 5-point Likert scale ranging from 1 (strongly disagree) to 5 (totally agree). Intention to use digital offerings was measured by asking, “I intend to use all digital offerings provided,” with answers rated on a 5-point Likert scale ranging from 1 (strongly disagree) to 5 (totally agree). These stage, intention, and attitude items were adapted and modified based on the health action process approach and have shown acceptable reliability and validity [50]. The perceived usefulness of digital offerings was measured by the item “I think digital training on rehabilitation goals is...,” with answers rated on a 5-point Likert scale ranging from 1 (not useful at all) to 5 (extremely useful). This item was adapted and modified based on the Technology Acceptance Model, which evaluates patients’ responses to health information technology [51] and has shown acceptable reliability and validity [50].

COVID-19 pandemic–related information was assessed regarding the participant’s job [50]; COVID-19 infection [43]; medical treatment and change in physical activity perspectives [43]. Work ability risk was measured by asking, “Do you feel that your ability to work is permanently endangered by the Corona pandemic?” The answers were rated on a 4-point Likert scale ranging from 1 (definitely not) to 4 (definitely). Status of losing one’s job was measured by asking, “Did you lose your job due to the Corona crisis?” Worries about losing a job was measured by asking, “Are you worried about your job due to the Corona crisis?” The answers were rated on a 5-point Likert scale ranging from 1 (no, not at all) to 5 (yes, completely). Financial impact was measured by asking, “Is the Corona crisis having a negative impact on your income?” The answers were rated on a 5-point Likert scale ranging from 1 (no, not at all) to 5 (yes, completely). Infection of COVID-19 was measured by asking, “Have you been infected with the coronavirus?” Fears of COVID-19 infection were measured by 3 items (eg, “How often do you fear being infected with the coronavirus?”), with answers rated on a 5-point Likert scale ranging from 1 (never) to 5 (always) and with a Cronbach α value of .85 in this study. Attitude to restriction rules was measured by asking, “I think the restrictions of the current visit regulations are appropriate.” Anxiety owing to news related to COVID-19 was measured by asking, “How often do you experience anxiety while following the news?” The answers were rated on a 5-point Likert scale ranging from 1 (never) to 5 (always). Medical treatment experience was measured by asking, “Have you (before rehab) or your loved ones received medical treatment since the beginning of the coronavirus outbreak?” Change in physical activity behavior was measured by asking, “Compared to before the outbreak of the coronavirus pandemic, have you become less or more physically active?” These aspects were analyzed separately.

### Statistical Analyses

Chi-square tests were performed to examine the differences between dropout patients and retained patients in terms of sociodemographic variables. To compare the dropout rate differences between the CG and the IGs, a chi-square test was conducted. Logistic regression analyses were used to assess what factors were related to survey dropout. An ANOVA was performed to test whether there was a difference between retained patients and dropout patients regarding the total time spent on answering the T1 survey questionnaire. Analyses were performed with SPSS software (version 27.0; IBM Corp).

## Results

### Sociodemographic Characteristics of Patients

A total of 2155 patients participated in the T1 web-based survey. If a patient started to fill in the T1 questionnaire but did not start filling in the T2 questionnaire, this patient was considered a study dropout. Of the 2155 patients, 1082 (50.21%) were categorized as dropouts in the T2 web-based survey, with 1073 (49.79%) participating in both the T1 and T2 web-based surveys. There were no significant differences regarding sex between the patients who dropped out after T1 and those who completed both web-based surveys (Table 1; *P*<.05). However, there were significant differences regarding age and education level: among patients aged ≥50 years, 53.72% (693/1290) of the patients participated in both surveys, whereas among those aged <50 years, 43.8% (376/858) of the patients participated in both surveys. Regarding education level, the retention rate was the lowest (20/66, 30%) among patients who had <11 years of schooling and the highest (337/593, 56.8%) among patients who had a university degree. Among the 2155 participants, there were 13 (0.6%) participants with missing data points regarding sex, 7 (0.32%) participants with missing data points regarding age, and 35 (1.62%) participants with missing data points regarding education status. The characteristics of participants with no missing data regarding these sociodemographic variables are shown in Table 1.

**Table 1:** Sociodemographic characteristics and differences between dropout patients and retained patients at baseline.

			Total, n (%)		Dropout patients, n (%)		Retained patients, n (%)		Chi-square (*df*)			*P* value	
**Sex**										0.3 (2)			.85
	Male	742 (34.64)^a^		373 (34.7)^b^		369 (34.58)^c^							
	Female	1397 (65.22)^a^		700 (65.12)^b^		697 (65.32)^c^							
	Intersex	3 (0.14)^a^		2 (0.19)^b^		1 (0.09)^c^							
**Age (y)**										24.6 (4)			<.001
	≤29	89 (4.14)^d^		53 (4.91)^e^		36 (3.37)^f^							
	30-39	271 (12.62)^d^		160 (14.83)^e^		111 (10.38)^f^							
	40-49	498 (23.18)^d^		269 (24.93)^e^		229 (21.42)^f^							
	50-59	984 (45.81)^d^		444 (41.15)^e^		540 (50.51)^f^							
	≥60	306 (14.25)^d^		153 (14.18)^e^		153 (14.31)^f^							
**Highest education level**										26.2 (3)			<.001
	≤11 y of schooling	66 (3.11)^g^		46 (4.31)^c^		20 (1.9)^h^							
	≥12 y of schooling	482 (22.74)^g^		264 (24.74)^c^		218 (20.7)^h^							
	Vocational training	979 (46.18)^g^		501 (46.95)^c^		478 (45.39)^h^							
	University degree	593 (27.97)^g^		256 (23.99)^c^		337 (32)^h^							

^a^N=2142.

^b^N=1075.

^c^N=1067.

^d^N=2148.

^e^N=1079.

^f^N=1069.

^g^N=2120.

^h^N=1053.

### Interventions and Diagnoses of Patients

In this study, 3 digital interventions were offered to the patients on a voluntary basis. Of the 2155 patients, 1855 (86.08%) participated in the digital training on rehabilitation goals, 260 (12.06%) participated in the digital group training for depression, and 277 (12.85%) participated in the informative digital training on the legal rights of people who are (severely) disabled. Patients in the CG (253/2155, 11.74%) did not participate in any of the 3 digital trainings. Patients who participated in 1 of the 3 digital trainings (1561/2155, 72.44%) were defined as IG 1, those who participated in 2 of the 3 digital trainings (192/2155, 8.91%) were defined as IG 2, and those who participated in all 3 digital trainings (149/2155, 6.91%) were defined as IG 3.

When comparing the dropout differences among these 4 groups, there were significant differences (*χ*^2^_3_=39.7; *P*<.001). The CG had the highest dropout rate of 68.4% (173/253), compared with IG 1 with 47.98% (749/1561), IG 2 with 50% (96/192), and IG 3 with 42.9% (64/149).

**Figure 1:**
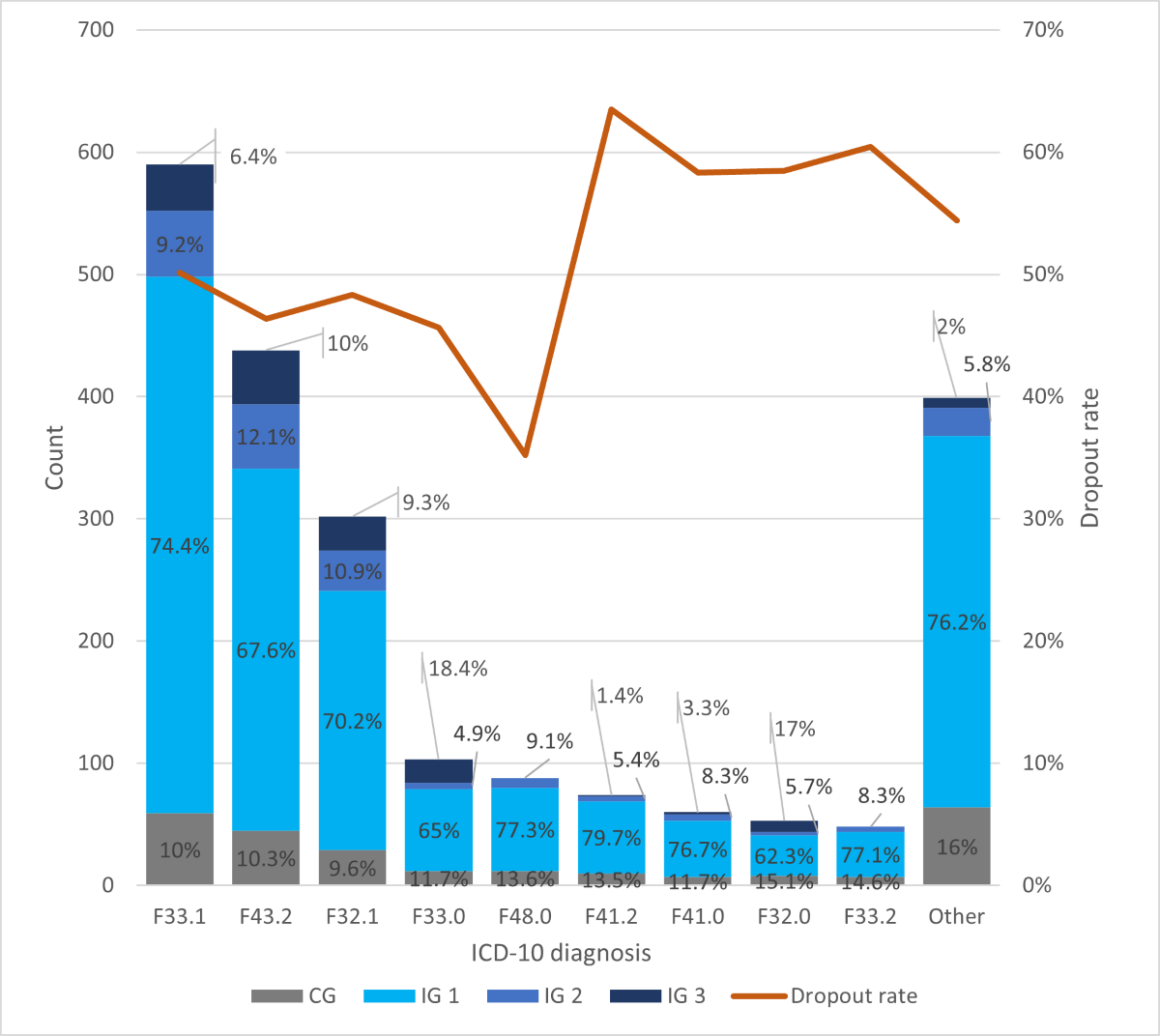
The percentages of participation in the 4 study groups and dropout rate among patients with different International Classification of Diseases, 10th Revision (ICD-10), diagnoses. The dropout rate within 1 diagnosis is the percentage of the number of dropout patients divided by the total number of patients with this diagnosis. CG: comparison group; F32.0: major depressive disorder, single episode, mild; F32.1: major depressive disorder, single episode, moderate; F33.0: major depressive disorder, recurrent, mild; F33.1: major depressive disorder, recurrent, moderate; F33.2: major depressive disorder, recurrent severe without psychotic features; F41.0: panic disorder; F41.2: mixed anxiety and depressive disorder; F43.2: adjustment disorder; F48.0: neurasthenia; IG: intervention group.

All patients received their diagnoses at baseline according to the International Classification of Diseases, 10th Revision (ICD-10), manual. The most common diagnoses were *major depressive disorder, recurrent, moderat*e (F33.1; 590/2155, 27.38%); *adjustment disorder* (F43.2; 438/2155, 20.32%); *major depressive disorder, single episode, moderate* (F32.1; 302/2155, 14.01%); *major depressive disorder, recurrent, mild* (F33.0; 103/2155, 4.78%); *neurasthenia* (F48.0; 88/2155, 4.08%); *mixed anxiety and depressive disorder* (F41.2; 74/2155, 3.43%); *panic disorder* (F41.0; 60/2155, 2.78%); *major depressive disorder, single episode, mild* (F32.0; 53/2155, 2.46%); and *major depressive disorder, recurrent severe without psychotic features* (F33.2; 48/2155, 2.23%). The other 87 types of diagnoses (each <35/2155, <1.62%) were summarized as “Other” (Figure 1).

The dropout rates (the dropout rate within 1 diagnosis was the percentage of the number of dropout patients divided by the total number of patients with this diagnosis) among patients with different ICD-10 diagnoses are shown in Figure 1. Patients with a diagnosis code of F41.2 had the highest dropout rate (47/74, 64%), followed by patients with a diagnosis code of F33.2 (29/48, 60%). Patients with a diagnosis code of F48.0 had the lowest dropout rate (31/88, 35%) compared with other diagnoses in this study (Figure 1). The percentages of participation in the 4 study groups among patients with different ICD-10 diagnoses are also shown in Figure 1.

### Dropout Predictors

To further investigate whether the research groups (model 0), and rehabilitation and sociodemographic variables (model 1), and physical and mental health–related variables at baseline (model 2), and digital use–related variables (model 3), and COVID-19 pandemic–related variables (model 4) were interrelated with study dropout at T2, logistic regression analyses were performed. This was performed using dummy coding for patients who remained in the study at T2 as 0 versus those who dropped out as 1.

Model 0 showed the comparisons between the IGs (IG 1, IG 2, and IG 3) and the CG and found that patients in the IGs were less likely to drop out of the study (IG 1 vs CG: odds ratio [OR] 0.37, 95% CI 0.26-0.54; *P*<.001; IG 2 vs CG: OR 0.52, 95% CI 0.31-0.87; *P*=.01; and IG 3 vs CG: OR 0.30, 95% CI 0.18-0.53; *P*<.001). This pattern was also found in the other 4 models (Multimedia Appendix 1). When considering sociodemographic variables, it was found that patients aged ≥50 years were less likely to drop out of the study than younger patients, and patients who had higher education levels (≥12 y of schooling, vocational training, or a university degree) were also less likely to drop out. When considering physical and mental health of patients, those who did not know whether they had underlying diseases were less likely to drop out than those who knew that they had underlying diseases. Patients who had a diagnosis of *adjustment disorder* (F43.2) as well as patients who had a diagnosis of *neurasthenia* (F48.0) were less likely to drop out than patients who had a diagnosis of *major depressive disorder, recurrent, moderate* (F33.1). When further considering the impact of patients’ digital use on study dropout, it was found that patients who had more use of health-related app and the internet were less likely to drop out of the study. Among these health-related apps, apps for “Corona-related content (eg, warnings about risk areas)” had the most frequent use—of the 2155 patients, 1148 (53.27%) reported this option—followed by “exercise and fitness (eg, pedometer)” reported by 46.82% (1009/2155) of the patients and “doctor search, appointment scheduling and contact with doctors” reported by 45.71% (985/2155) of the patients. When further considering the interrelation with the COVID-19 pandemic on study dropout, it was found that patients who feel their work ability had lower risk to be permanently endangered by COVID-19 were less likely to drop out of the study. Moreover, patients who lost their job as a result of the COVID-19 crisis were less likely to drop out of the study than those who maintained their job; furthermore, patients who had not been infected by COVID-19 were less likely to drop out of the study.

### Time Spent on the Survey

To test whether there was a difference between retained patients and dropout patients in the total time spent on answering the T1 questionnaires, an ANOVA was conducted. There was no significant difference in the total time spent (*F*_1,2059_=3.49; *P*=.06) between retained patients (mean 22.0, SD 9.50 min; range 8.3-65.6 min) and dropout patients (mean 22.8, SD 10.08 min; range 3.0-67.3 min). Among the 1082 dropout patients, there were 53 (4.9%) participants with missing data regarding time spent on the survey, and among the 1073 retained patients, there were 41 (3.82%) participants with missing data. If the web page that the patients were working on was closed and then reopened (eg, owing to a power interruption, internet connection issue, or patients closing the website intentionally or accidentally), the time spent on the questionnaire would not have been recorded correctly, and thus they would be considered missing data points.

## Discussion

### Principal Findings

This study investigated the factors that relate to whether participants (ie, patients who participated in a psychosomatic rehabilitation) drop out of a web-based study by focusing on sociodemographic factors, voluntary interventions, physical and mental health, digital use for health and rehabilitation, and COVID-19 pandemic–related information of participants. In conformity with social cognitive theory, the results revealed that personal and external factors, including age, education level, diagnoses of mental disorders, the digital training involved, health-related digital use behaviors, whether participants were infected with COVID-19, and whether participants lost a job owing to the COVID-19 pandemic were all possible predictors of web-based study dropout.

### Mental and Behavioral Disorder Diagnoses as Predictors

One of the main findings was that patients with different disorder diagnoses showed different dropout rates in this study. Patients with a diagnosis of *mixed anxiety and depressive disorder* or *major depressive disorder, recurrent severe without psychotic features* were more likely to drop out of the study than other patients, such as those diagnosed with *adjustment disorder* or *neurasthenia*. This shows that the motivational deficits and concentration issues primarily associated with *major depressive disorder, recurrent severe without psychotic features* and *mixed anxiety and depressive disorder* may provide an explanation for why these individuals dropped out from the study, whereas patients with other diagnoses that do not necessarily fall under the umbrella of affective disorders rather were insignificant in terms of predicting dropout.

These findings added important comparative information among different diagnoses to previous studies on the dropout investigation of 1 or a few diagnosed mental disorders, such as depressive disorder [1] and adjustment disorder [52]. Although web-based interventions have been proven to be effective and convenient and saved costs in many studies [7,30], it has also been found that they have a high dropout rate, especially when conducting self-help interventions [52]. Therefore, for web-based interventions that involve patients with mental disorders, especially patients with depressive disorders, it is suggested to offer more support via consultants [30], as well as provide feedback and reminders for engaging in the intervention [53] and in-app mood monitoring [1]. This could help participants overcome the motivational deficits with regard to completing the survey.

### COVID-19 Pandemic Correlates as Predictors

Patients who reported having been infected with COVID-19 had a greater risk of dropping out of this study than patients who had not been infected. On the one hand, it is possible that some patients who had been infected might subsequently experience post–COVID-19 condition symptoms [54], which increases the chances of patients dropping out because they were experiencing symptoms such as fatigue and shortness of breath [55]. This is important because the rates of infection are still rising in the population, and, according to different studies, approximately one-third of all people infected with COVID-19 experience post–COVID-19 condition symptoms [54]. On the other hand, previous studies also found that infection with COVID-19 predicted a decrease in mental health [56], making more effective mental health services essential for these patients. Dropping out from web-based studies might lead to losing the opportunity to obtain digital training and web-based support for this population. How to prevent such a contradiction was not explicitly addressed in this study and calls for future investigation, especially in research on post–COVID-19 condition and when testing tailored interventions in experimental designs.

Another COVID-19 pandemic–related predictor of the dropout rate was that patients who lost their job as a result of the COVID-19 crisis were more likely to remain in the study than those who maintained their jobs. This could be because the latter were more time restricted, and the former were more likely to have more time as well as perceive a higher need to retrieve any useful information and attention. Previous studies have found that patients might drop out of psychological services owing to logistical barriers such as time limitations [57]. A job loss, in the long term, might result in financial troubles, which was found to be related to dropout [58]. In this study, the financial impact of COVID-19 crisis was not a significant predictor of dropout. This might be because the data collection was conducted at a relatively early time during the COVID-19 pandemic, and the financial impact has not been fully revealed. Patients who lost their job might have had more time to remain in the study. Further research is suggested to explicitly assess this point by providing data comparison from different time periods of the COVID-19 pandemic.

### Digital Use as Predictors

It was also found that patients who reported more use of health-related app and the internet were more likely to remain in the study. This could be because patients who were more concerned and focused on their health could have wanted to participate in further health research that could foster or lead to an improvement in their health. Moreover, prior experience, a positive attitude, and beliefs about digital use for health were related to participation in digital interventions. This finding can be explained by the theoretical model of the health action process approach, which explains how psychological factors predict behavior, and is in line with previous studies [16,59-61].

The finding that patients who have higher education levels were more likely to remain in the study was also consistent with previous studies [7,24,62]. Patients with a relatively lower education level were more likely to drop out, possibly because they do not feel that the study matched with their needs. In addition, as there is perhaps a low need for digital technology in their job and daily life, they could lack the skills and experience needed to use digital offerings; the lack of self-reflection and eloquence needed could have made it difficult to take advantage of digital offerings [7]. It is suggested in the future to provide digital skills training to participants before they participate in web-based interventions to reduce such inequalities in using digital offerings and decrease dropout rates accordingly, which will improve participation.

### Sociodemographics as Predictors

In this study, younger patients were found to have a greater risk of dropping out than older patients. This is consistent with previous studies investigating dropouts among patients with mental disorders [7,21,63], but it is contradictory to other studies with older participants on average [12] or studies with participants who have a physical disease [64]. When referring to the findings of this study, it is necessary to also consider that the mode of age was 50 to 59 years, and more than half of the patients reported that they did not have an underlying disease (1123/2139, 52.5%) or that they were not disabled (1830/2139, 85.55%). Older patients who remained in this study might have done so because they were not having a very difficult time in terms of their health status in a way that impaired their ability to remain in the study, or they might have had less of a time conflict than younger participants [7].

### Study Designs as Predictors

In this study, patients in the CG had the highest dropout rate compared with the other study groups. This finding is contrary to some studies that reported a higher dropout rate in the IG than in the CG [1,32]. These studies found that the extra tasks imposed burdens on participants in the IGs and hence made them more likely to drop out. Another aspect that was different from these randomized controlled trial studies was that the method of grouping in this study was according to the number of digital trainings that patients participated in voluntarily rather than random assignment. If 3 digital trainings were considered an overload by some patients, they could choose 2, 1, or no trainings to participate in. This might relate to the lower dropout rates in the IGs. Patients in the CG who did not participate in any of the digital trainings could have been less motivated or limited by their health status or lack of digital skills. These factors might also be related to their dropping out from the study.

The time taken to complete the web-based questionnaire was recorded because this could have provided insight into the different issues as well. No significant difference was found between retained patients and dropout patients regarding the total time spent on answering the T1 survey questionnaire. Therefore, the length of the questionnaire was not regarded as a factor that relates to dropout.

Although previous studies have found higher dropout rates in large-sample studies, it was also found that providing feedback to participants can decrease the dropout rate [1,32,65]. Participants might have had more motivation from the feedback provided by research assistants or feedback supported by technology (eg, the use of chatbots, which can save much time for research assistants, especially in large-sample studies), enabling them to complete the subsequent study surveys.

### Limitations

There are several limitations that should be considered. One limitation is that the dropout rate investigation was not explicitly planned in the study protocol; therefore, no preventive measures were applied. There were no explicit variables that measured the reason why patients dropped out of the study. In addition, some factors, such as personal values, that have potential influence on dropouts were not included in this study [66]. Future studies should include further variables, such as individual differences in personality or values, to increase the predictive power and to control potential third-variable problems.

A further limitation that needs to be discussed is that the digital trainings were conducted before and during patients’ rehabilitation stay at psychosomatic rehabilitation clinics, where approximately 5.99% (129/2155) of the patients were found to have a shorter rehabilitation stay than planned. Possible reasons why patients might end their rehabilitation stay earlier than planned include the following: (1) patients and health care professionals may come to a shared understanding that the intended rehabilitation is not suitable for the health-related needs of the patient; (2) patients might experience a poor physical health status in general that limited their rehabilitation completion; or (3) patients might experience unexpected events, such as being infected with COVID-19. These could also be the reasons for patients dropping out of the study. Future longitudinal research is suggested to consider sending a follow-up message to patients who did not appear at the planned measurement time points, but the response rate would likely be low. In addition, more interactive supports are suggested to be provided to those patients who have a high risk of dropout.

Furthermore, because the data were collected at 4 psychosomatic clinics, it is possible that the somatic aspects are a confounding factor for mental health. As a result, the logistic regression analysis might suffer from multicollinearity. Moreover, the sample of psychosomatic rehabilitation patients might not represent general populations. This point remains to be evaluated in further research that includes data from other clinics and general populations. Another concern regarding this sample is that this study was conducted in Germany, where the main rehabilitation goal is social and work participation, and the psychosomatic rehabilitation patients were mostly those with access to pension insurance funds. Further studies conducted in other countries that recruit participants from different backgrounds are expected to replicate the findings in this study to compare the concerns regarding dropout in psychosocial web-based studies.

### Conclusions

The findings from this study indicated that different factors such as patient sociodemographics, physical and mental health, digital use, COVID-19 pandemic correlates, and study design can correlate with the dropout rate in web-based interventions. To decrease the dropout rate, these possible predictors of web-based study dropout should be taken into account, and more support should be given to participants who have a high risk of dropping out of web-based studies: young patients, patients who are less educated, patients with a depressive disorder, patients who have few health-related digital experiences, and patients infected with COVID-19.

## Appendix

Multimedia Appendix 1 Logistic regression models predicting study dropout with dummy coding for patients who remained in the study at T2 as 0 versus those who dropped out as 1.

## Abbreviations

CG: comparison group
ICD-10: International Classification of Diseases, 10th Revision
IG: intervention group
OR: odds ratio
PHQ-4: Patient Health Questionnaire-4
PSS-4: 4-item version of the Perceived Stress Scale

## Data Availability

The data for this study will not be publicly available owing to data protection guidelines. The data will be available upon reasonable request to the corresponding author.
