# Supplementary material for: Predictors of Dropout Among Psychosomatic Rehabilitation Patients During the COVID-19 Pandemic: Secondary Analysis of a Longitudinal Study of Digital Training
Source: J Med Internet Res. 2023 Nov 27;25:e43584. doi: 10.2196/43584 (PMC10683786; doi:10.2196/43584)
Supplement: Multimedia Appendix 1 [file jmir_v25i1e43584_app1.docx]

**Multimedia Appendix 1.** Logistic regression models predicting study dropout with dummy coding for patients who remained in the study at T2 as 0 versus those who dropped out as 1.

| Variables | | | Model 1 | | Model 2 | | Model 3 | | Model 4 | |
| --- | --- | --- | --- | --- | --- | --- | --- | --- | --- | --- |
|  | | | OR^a^ (95% CI) | *P* value | OR (95% CI) | *P* value | OR (95% CI) | *P* value | OR (95% CI) | *P* value |
|  | | | | | | | | | | |
| **Research groups** | | | | | | | | | | |
|  | CG^b^ | | 1 | N/A^d^ | 1 | N/A | 1 | N/A | 1 | N/A |
|  | IG^c^ 1 | | 0.36 (0.25-0.53) | <.001 | 0.35 (0.24-0.52) | <.001 | 0.37 (0.25-0.55) | <.001 | 0.37 (0.25-0.56) | <.001 |
|  | IG 2 | | 0.52 (0.31-0.88) | .02 | 0.51 (0.30-0.88) | .01 | 0.52 (0.30-0.90) | .019 | 0.52 (0.30-0.90) | .019 |
|  | IG 3 | | 0.31 (0.18-0.55) | <.001 | 0.30 (0.17-0.55) | <.001 | 0.33 (0.18-0.59) | <.001 | 0.34 (0.18-0.61) | <.001 |
| **Rehabilitation** | | | | | | | | | | |
|  | Rehabilitation goals | | 1.32 (0.97-1.78) | .08 | 1.27 (0.93-1.74) | .14 | 1.42 (1.03-1.96) | .04 | 1.37 (0.98-1.90) | .07 |
|  | Days of rehabilitation stay | | 0.98 (0.96-0.99) | .01 | 0.98 (0.96-0.99) | .008 | 0.98 (0.96-0.99) | .01 | 0.98 (0.96-0.99) | .016 |
| **Sociodemographic variables** | | | | | | | | | | |
|  | Sex (male=1) | | 0.88 (0.70-1.13) | .33 | 0.90 (0.70-1.15) | .40 | 0.96 (0.74-1.23) | .72 | 0.97 (0.74-1.27) | .81 |
|  | **Age (years)** | | | | | | | | | |
|  |  | ≤29 | 1 | N/A | 1 | N/A | 1 | N/A | 1 | N/A |
|  |  | 30-39 | 0.78 (0.37-1.63) | .51 | 0.88 (0.41-1.86) | .74 | 0.80 (0.37-1.70) | .56 | 0.76 (0.35-1.64) | .48 |
|  |  | 40-49 | 0.62 (0.31-1.25) | .18 | 0.70 (0.34-1.43) | .33 | 0.63 (0.30-1.30) | .21 | 0.54 (0.26-1.14) | .10 |
|  |  | 50-59 | 0.38 (0.20-0.76) | .006 | 0.45 (0.22-0.91) | .03 | 0.38 (0.19-0.77) | .008 | 0.33 (0.16-0.68) | .003 |
|  |  | ≥60 | 0.53 (0.25-1.10) | .09 | 0.62 (0.29-1.32) | .21 | 0.51 (0.23-1.09) | .08 | 0.41 (0.19-0.90) | .03 |
|  | **Educational status** | | | | | | | | | |
|  |  | ≤11 years of schooling | 1 | N/A | 1 | N/A | 1 | N/A | 1 | N/A |
|  |  | ≥12 years of schooling | 0.33 (0.15-0.73) | .006 | 0.34 (0.15-0.77) | .01 | 0.35 (0.15-0.80) | .01 | 0.35 (0.15-0.82) | .02 |
|  |  | Vocational training | 0.33 (0.15-0.71) | .005 | 0.36 (0.16-0.79) | .01 | 0.37 (0.17-0.84) | .02 | 0.37 (0.16-0.86) | .02 |
|  |  | University degree | 0.24 (0.11-0.52) | <.001 | 0.26 (0.11-0.58) | <.001 | 0.27 (0.12-0.62) | .002 | 0.28 (0.12-0.64) | .003 |
| **Physical and mental health** | | | | | | | | | | |
|  | Regular physical activity (no=1) | | N/A | N/A | 0.80 (0.62-1.02) | .07 | 0.84 (0.66-1.09) | .19 | 0.87 (0.67-1.13) | .31 |
|  | Enough vegetables and fruits intake (no=1) | | N/A | N/A | 0.78 (0.58-1.04) | .09 | 0.80 (0.60-1.08) | .14 | 0.80 (0.59-1.08) | .15 |
|  | **BMI (kg/m^2^)** | | | | | | | | | |
|  |  | Normal weight | N/A | N/A | 1 | N/A | 1 | N/A | 1 | N/A |
|  |  | Underweight | N/A | N/A | 1.19 (0.49-2.92) | .70 | 1.16 (0.46-2.88) | .76 | 1.03 (0.41-2.60) | .95 |
|  |  | Overweight | N/A | N/A | 1.04 (0.77-1.40) | .80 | 1.07 (0.79-1.44) | .67 | 1.10 (0.78-1.46) | .63 |
|  |  | Obese | N/A | N/A | 0.96 (0.71-1.29) | .76 | 0.98 (0.73-1.32) | .90 | 0.97 (0.71-1.31) | .82 |
|  | **Underlying diseases** | | | | | | | | | |
|  |  | “Yes” | N/A | N/A | 1 | N/A | 1 | N/A | 1 | N/A |
|  |  | “No” | N/A | N/A | 0.96 (0.75-1.24) | .77 | 0.92 (0.71-1.20) | .55 | 0.94 (0.72-1.22) | .62 |
|  |  | “Don’t know” | N/A | N/A | 0.68 (0.41-1.11) | .12 | 0.64 (0.39-1.06) | .08 | 0.59 (0.35-0.99) | .048 |
|  | Pass issued to persons who are disabled (no=1) | | N/A | N/A | 0.97 (0.67-1.40) | .85 | 0.96 (0.66-1.40) | .83 | 0.95 (0.65-1.39) | .77 |
|  | Perceived loneliness | | N/A | N/A | 1.08 (0.92-1.26) | .34 | 1.07 (0.91-1.24) | .44 | 1.02 (0.87-1.20) | .79 |
|  | Perceived stress | | N/A | N/A | 0.97 (0.91-1.03) | .28 | 0.96 (0.90-1.02) | .20 | 0.95 (0.89-1.02) | .14 |
|  | Perceived depression and anxiety | | N/A | N/A | 1.01 (0.96-1.06) | .75 | 1.01 (0.96-1.07) | .74 | 1.01 (0.95-1.06) | .83 |
|  | **ICD-10^e^ diagnoses** | | | | | | | | | |
|  |  | F33.1^f^ | N/A | N/A | 1 | N/A | 1 | N/A | 1 | N/A |
|  |  | F43.2^g^ | N/A | N/A | 0.66 (0.47-0.93) | .02 | 0.65 (0.46-0.92) | .02 | 0.63 (0.45-0.90) | .01 |
|  |  | F32.1^h^ | N/A | N/A | 0.73 (0.50-1.06) | .10 | 0.73 (0.50-1.07) | .11 | 0.71 (0.48-1.05) | .09 |
|  |  | F33.0^i^ | N/A | N/A | 0.67 (0.38-1.18) | .16 | 0.65 (0.37-1.15) | .14 | 0.63 (0.35-1.14) | .13 |
|  |  | F48.0^j^ | N/A | N/A | 0.47 (0.26-0.84) | .01 | 0.49 (0.27-0.87) | .02 | 0.45 (0.25-0.83) | .01 |
|  |  | F41.2^k^ | N/A | N/A | 0.93 (0.48-1.80) | .83 | 0.98 (0.50-1.92) | .96 | 0.90 (0.46-1.79) | .77 |
|  |  | F41.0^l^ | N/A | N/A | 0.64 (0.30-1.34) | .24 | 0.63 (0.29-1.34) | .23 | 0.67 (0.31-1.45) | .31 |
|  |  | F32.0^m^ | N/A | N/A | 1.30 (0.63-2.68) | .48 | 1.24 (0.60-2.57) | .57 | 1.10 (0.52-2.31) | .81 |
|  |  | F33.2^n^ | N/A | N/A | 1.74 (0.55-5.53) | .35 | 1.61 (0.50-5.20) | .43 | 1.35 (0.41-4.46) | .62 |
|  |  | Other | N/A | N/A | 0.80 (0.55-1.17) | .25 | 0.80 (0.55-1.17) | .24 | 0.77 (0.52-1.13) | .19 |
| **Digital use** | | | | | | | | | | |
|  | Health-related digital use behavior | | N/A | N/A | N/A | N/A | 0.88 (0.82-0.94) | <.001 | 0.88 (0.82-0.95) | <.001 |
|  | Attitude toward digital offerings | | N/A | N/A | N/A | N/A | 0.91 (0.75-1.09) | .31 | 0.91 (0.75-1.10) | .32 |
|  | Intention to use digital offerings | | N/A | N/A | N/A | N/A | 1.07 (0.89-1.27) | .49 | 1.07 (0.89-1.29) | .47 |
|  | Stage of health app use_app 1 | | N/A | N/A | N/A | N/A | 1.02 (0.92-1.13) | .73 | 1.03 (0.92-1.14) | .64 |
|  | Stage of health app use_app 2 | | N/A | N/A | N/A | N/A | 0.99 (0.88-1.12) | .92 | 0.99 (0.87-1.12) | .89 |
|  | Perceived usefulness of digital offers | | N/A | N/A | N/A | N/A | 0.89 (0.74-1.06) | .20 | 0.91 (0.75-1.09) | .29 |
| **COVID-19 pandemic** | | | | | | | | | | |
|  | Work ability risk | | N/A | N/A | N/A | N/A | N/A | N/A | 1.22 (1.04-1.44) | .02 |
|  | Job loss (no=1) | | N/A | N/A | N/A | N/A | N/A | N/A | 0.33 (0.13-0.83) | .02 |
|  | Job worries | | N/A | N/A | N/A | N/A | N/A | N/A | 1.03 (0.91-1.16) | .67 |
|  | Financial impact | | N/A | N/A | N/A | N/A | N/A | N/A | 0.92 (0.81-1.03) | .14 |
|  | **Infected with COVID-19** | | | | | | | | | |
|  |  | “No” | N/A | N/A | N/A | N/A | N/A | N/A | 1 | N/A |
|  |  | “Don’t know” | N/A | N/A | N/A | N/A | N/A | N/A | 1.11 (0.79-1.57) | .56 |
|  |  | “Yes” | N/A | N/A | N/A | N/A | N/A | N/A | 2.28 (1.08-4.81) | .03 |
|  | Infection fears | | N/A | N/A | N/A | N/A | N/A | N/A | 0.93 (0.79-1.09) | .35 |
|  | Anxiety owing to news related to COVID-19 | | N/A | N/A | N/A | N/A | N/A | N/A | 1.09 (0.94-1.26) | .25 |
|  | Restriction rules (disagree=1) | | N/A | N/A | N/A | N/A | N/A | N/A | 0.79 (0.58-1.09) | .15 |
|  | **Medical treatment** | | | | | | | | | |
|  |  | “Yes” | N/A | N/A | N/A | N/A | N/A | N/A | 1 | N/A |
|  |  | “No” | N/A | N/A | N/A | N/A | N/A | N/A | 1.27 (0.91-1.77) | .16 |
|  |  | “Don’t know” | N/A | N/A | N/A | N/A | N/A | N/A | 1.71 (0.87-3.34) | .12 |
|  | **Physical activity change** | | | | | | | | | |
|  | Less | | N/A | N/A | N/A | N/A | N/A | N/A | 1 | N/A |
|  | Constant | | N/A | N/A | N/A | N/A | N/A | N/A | 0.90 (0.69-1.17) | .42 |
|  | More | | N/A | N/A | N/A | N/A | N/A | N/A | 0.73 (0.52-1.03) | .08 |

Model 1, Nagelkerke *R*^2^=.08; Model 2, Nagelkerke *R*^2^=.12; Model 3, Nagelkerke *R*^2^=.14; Model 4, Nagelkerke *R*^2^=.16.

^a^OR: odds ratio.

^b^CG: comparison group.

^c^IG: intervention group.

^d^N/A: not applicable.

^e^ICD-10: International Classification of Diseases, 10^th^ Revision.

^f^F33.1: *major depressive disorder, recurrent, moderat*e.

^g^F43.2: *adjustment disorder.*

^h^F32.1: *major depressive disorder, single episode, moderate.*

^i^F33.0: *major depressive disorder, recurrent, mild*.

^j^F48.0: *neurasthenia*.

^k^F41.2: *mixed anxiety and depressive disorder*.

^l^F41.0: panic disorder.

^m^F32.0: *major depressive disorder, single episode, mild*.

^n^F33.2: *major depressive disorder, recurrent severe without psychotic features*.
